# The role of eco-anxiety in the presentation of bulimia nervosa: a case report

**DOI:** 10.1186/s40337-024-01118-5

**Published:** 2024-10-21

**Authors:** Cristin D. Runfola, Debra L. Safer

**Affiliations:** Department of Psychiatry and Behavioral Sciences, Stanford University, 401 Quarry Dr, Stanford, CA 94304 USA

**Keywords:** Eating disorder, Bulimia nervosa, Eco-anxiety, Eco-concerns, Climate change, Treatment, CBT-E

## Abstract

**Background:**

Despite a growing literature demonstrating the significant impacts of climate change on mental health, research is urgently needed to investigate how climate change-related concerns may contribute to the development, exacerbation, or re-emergence of eating disorders, as well as affect the effectiveness of existing interventions. This case report contributes to this scant knowledge base by offering empirical evidence for how responses to climate change can influence eating disorder symptoms and, importantly, limit the effectiveness of evidence-based treatments such as Cognitive Behavior Therapy-Enhanced (CBT-E).

**Case presentation:**

A 24-year-old female graduate student studying environmental science presented to a specialized eating disorder clinic with worsening bulimia nervosa. Her symptoms initially improved with CBT-E; however, after three months, concerns about food waste significantly impeded further progress. The therapist, identifying symptoms of eco-anxiety, adapted standard CBT-E strategies to include psychoeducation about eco-anxiety, cognitive restructuring of beliefs about food waste and other eating-related eco-concerns, relevant exposures related to such concerns, and problem-solving to increase social support. These adaptations led to resumed progress, with the patient achieving nutritional adequacy by treatment end (38 sessions) and maintaining treatment gains through one year follow-up.

**Conclusions:**

To our awareness, this is the first case report on the co-occurrence of eco-anxiety and eating disorders. This case underscores the importance of screening for concurrent eco-anxiety, suggests ways in which eating disorders and eco-anxiety can influence one another longitudinally, describes how coexisting eco-anxiety can limit standard CBT-E’s effectiveness, and provides examples of successful treatment adaptations tailored to address eco-anxiety-related concerns.

## Background

Eating disorders, which often begin in adolescence and young adulthood, are severe and debilitating diagnoses. For example, anorexia nervosa is associated with one of the highest mortality rates of any psychiatric illness [1]. Due to the severity of these disorders, early detection and intervention are crucial. This process includes being alert to the ways in which societal and cultural factors can influence their onset and presentation. Recently, clinicians treating patients with eating disorders witnessed firsthand the impact of societal stressors during the COVID-19 pandemic when clinics reported a sharp increase (up to 40%) in demand for services [2]. This surge encompassed patients with new onset symptoms, exacerbation of existing symptoms, or re-emergence of previously resolved symptoms. Patients described symptoms associated with the strain of limited access to food (i.e., food insecurity), changes to physical activity, worsened mood (e.g., COVID-19 related fears, anxiety, stress), disruptions in routine, and confinement within triggering environments [2]. Understanding these pandemic-specific triggers was key to adapting current evidence-based treatments to maintain their effectiveness.

Not only are future pandemics more likely due to the climate crisis [3] but rising global temperatures will also increase the frequency and intensity of extreme weather events (e.g., wildfires, hurricanes, droughts, heat-waves), which have well-documented mental health consequences. These consequences include elevated rates of anxiety disorders, mood disorders, acute stress reactions, post-traumatic stress disorders, aggression, suicidal ideation, and suicide [4].

Another climate-change impact is a relatively newly-described syndrome, *eco-anxiety*, or the fear of environmental doom [5]. Individuals with eco-anxiety experience a variety of symptoms across cognitive (e.g., catastrophic thoughts and obsessive rumination about the future), emotional (e.g., fear, dread, anger, grief, guilt), behavioral (e.g., difficulty concentrating, interrupted sleep), and physiological (e.g., panic) domains [6]. These symptoms range from normal reactions to climate change threats to more intense symptoms that impair functioning. Although eco-anxiety is not a diagnosable mental health disorder, more severe presentations can overlap with mental health disorders.

Evidence indicates that young people are particularly vulnerable to eco-anxiety. In a global survey of 10,000 adolescents and young adults (aged 16–25 years), Hickman and colleagues [7] reported that 84% were at least moderately worried about climate change, 59% were very or extremely worried, and 75% thought the future was frightening. Many respondents expressed negative emotions, with more than 50% reporting each of the following: feeling afraid, sad, anxious, angry, powerless, helpless, and guilty [7]. Nearly half of the participants reported that their climate change worries adversely affected their daily lives and functioning in terms of eating, concentrating, work, school, sleeping, spending time in nature, playing, having fun, and/or relationships.

Late adolescence and young adulthood are recognized as vulnerable periods for the development of disordered eating [8] and other mental health syndromes [9]. The emergence of eco-anxiety in response to climate change can contribute additional stress during these critical years. While research on the mental health impacts of climate change is growing [10], studies specifically investigating the relationship between climate change and eating disorders remain sparse. In one of the few papers to address this topic, Rodgers and colleagues [11] emphasize the urgent need for research to better understand the specific ways in which climate change and associated eco-anxiety may: (1) increase the risk for eating disorders; (2) exacerbate or cause the re-emergence of eating disorder symptoms in individuals already diagnosed; and/or (3) limit the efficacy of existing interventions.

Related to the question of how climate change and associated eco-anxiety might increase risk for eating disorders is that some individuals, in response to concerns about the planet and the desire to reduce their carbon footprint, may choose to adopt a plant-based diet. For example, a survey conducted by the International Food Information Council (IFIC) in 2024 [12] found that 26% of American adults cited environmental concerns as a significant reason for following a vegetarian, vegan, or plant-based diet. This choice can have health implications, with well-established associations between a vegan/vegetarian diet and eating disorders [13]. Yet, as Moreno and colleagues [13] make clear, studies to date are largely cross-sectional and do not specify causality. In other words, more research is needed to help determine whether following a plant-based diet increases the risk of an eating disorder or whether individuals with eating disorders are attracted to restricted vegan/vegetarian diets to subconsciously or consciously disguise their dieting and lose weight.

To identify eating behaviors related to climate change concerns, Qi and colleagues [14] developed and validated the Eating-Related Eco-Concern Questionnaire (EREC). This 10-item scale measures eco-focused eating behaviors, such as such as avoiding meat, limiting food waste, and avoiding food with excess packaging. Qi et al. [14] investigated the relation between EREC scores and a validated measure of disordered eating, the Eating Disorder Examination-Questionnaire (EDE-Q) [15]. EREC scores were significantly associated with the EDE-Q global score and shape concern subscale. However, these associations became nonsignificant after excluding individuals who exceeded EDE-Q cut-off values for possible disordered eating. This result suggests that eco-related eating concerns can be objectively measured and differentiated from eating disorder concerns. In other words, individuals may have high eco-related eating concerns without significant eating disorder issues (e.g., low EDE-Q scores). Nonetheless, discerning the motivations behind eco-related eating behaviors among individuals with higher EDE-Q scores may pose challenges, given the high associations found between the EREC and EDE-Q global score and shape concern subscale. The authors stress the need for further investigation to clarify the directionality of this association. Such cross-sectional findings leave open the possibility that individuals who change their eating behaviors due to eco-concerns might be more likely to subsequently develop eating disorders, or individuals with existing eating disorders might be more likely to further modify their eating behaviors due to eco-concerns.

The following case report is presented given the dearth of studies on the association between climate change and eating disorders, as clinical case material can offer insight into potential mechanisms underlying disorders and, therefore, provide a useful starting point in designing future studies. Specifically, this case report offers assessment details regarding the longitudinal development of both climate change and eating disorder concerns as well as clinical data regarding how current treatments for eating disorders may need to be adapted when patients with eating disorders present with eco-anxiety. Lessons learned from the current case report are particularly timely given the absence of randomized controlled studies evaluating eco-anxiety interventions and data available for only two non-randomized treatments [16, 17]. To our knowledge, no studies, including case reports, have described the treatment of eco-anxiety when comorbid with an eating disorder.

## Case presentation

A 24-year-old heterosexual mixed-race cisgender female PhD student studying environmental science sought treatment at a specialized outpatient eating disorder clinic due to symptoms impacting her quality of life. She completed a thorough semi-structured psychological evaluation and began psychotherapy with one author (CDR), a licensed clinical psychologist with expertise in eating disorders. The patient consented to treatment and subsequently to the use of her de-identified case material in scientific publications. Stanford University School of Medicine’s Institutional Review Board (IRB) deemed ethical review unnecessary for this case report.

### Relevant eating disorder history and presenting symptoms

The patient developed anorexia nervosa-restricting type during adolescence. During her freshman year of college, as a long-distance runner, she began experiencing binge eating and purging behaviors. Bulimia nervosa was formally diagnosed her senior year of college. Although, at that time, she briefly engaged in therapy with an eating disorder sports psychologist, she found therapy unhelpful due to being “in denial” of her disorder. Her symptoms ameliorated after stopping collegiate cross-country but worsened upon relocating to a new city for graduate school, leading her to seek help.

At initial evaluation, she met DSM-5-TR criteria for bulimia nervosa, moderate-severe, alongside major depressive disorder, moderate, recurrent with a seasonal pattern and generalized anxiety disorder [18]. She exhibited temperament traits of clinical perfectionism, achievement orientation, obsessionality, sensitivity to criticism and errors, altered reward sensitivity, harm avoidance, cognitive rigidity, and weak central coherence. She denied any past trauma. Her prior medical history included amenorrhea, osteopenia, and multiple stress fractures. At initial evaluation, she had bradycardia, iron deficiency, and low vitamin D. Cognitively, preoccupation with her weight and shape impeded her academic focus. Socially, she struggled to eat around others leading to social isolation from friends and her romantic partner. Distressed by her worsening eating disorder symptoms and their adverse impacts on her physical, cognitive, and psychosocial functioning, she was highly motivated for treatment.

Based on questions adapted from the Eating Disorders Examination [19], a well-validated measure of the presence and severity of eating disorder symptoms, the patient endorsed objective binge eating 3–4 days per week, near daily subjective binge eating, daily excessive, driven and compensatory exercise, and purging (self-induced vomiting) 2–3 days per week, on average, over the past three months. Purging behavior was motivated by desires to control her weight/shape and alleviate physical discomfort. She also endorsed significant dietary restrictions, including meal skipping, avoidance of specific foods (e.g., animal products, high sugar and high carbohydrate foods such as baked goods), and reduced portion sizes driven by desires for eating control, performance concerns, and pursuit of an athletic ideal.

When questioned about her reasons for avoiding specific foods, she explained that she became vegan her freshman year of college, two years after the onset of her eating disorder. Further elaboration revealed her motivation originated from class work undertaken for her environmental studies major, which led to a desire to reduce her carbon footprint. She expressed uncertainty regarding the influence of her concerns about shape/weight on her decision to adopt a vegan diet.

The patient’s height measured at 5’7’’. Her weight of 135 lbs. (21.1 kg/m^2^ BMI) was above her lowest weight of 110 lbs. (BMI 17.2 kg/m^2^) during high school in the context of anorexia nervosa and below her highest weight of 145 lbs. during college. She endorsed unstable body image and engaged in frequent body checking behaviors, such as weighing herself daily. Her ideal body image was characterized as “strong and skinny,” and she was concerned with body composition, preferring muscularity. In addition to academic achievement, both her shape and weight highly influenced her self-worth.

### Treatment course with eco-anxiety adaptations

Treatment involved 38 weekly 55-minute sessions of telehealth-delivered individual psychotherapy delivered via Zoom, a Health Insurance Portability and Accountability Act-approved platform. Therapy aimed to address the core maintenance factors of the eating disorder, as delineated in Fairburn’s Cognitive Behavioral Therapy-Enhanced (CBT-E) manual [20]. Treatment also included regular medical monitoring with a physician specialized in eating disorders and a few sessions of nutritional counseling provided by a specialized eating disorder sports dietitian.

Initially, the patient rapidly improved. She was able to reinstate regular eating patterns as well as reduce binge eating and purging behaviors quickly during the first month of treatment. This progress was partly because the psychoeducation she received about the rationale for regular eating made sense to her and partly because of her motivation to address bradycardia and avoid medical hospitalization. Throughout the second month, she continued to enhance food variety and portion sizes through the implementation of standard cognitive and behavioral interventions outlined in CBT-E.

However, by the third month, escalating concerns about climate change, triggered by coursework, began to interfere with treatment. Employing Socratic questioning and using data from her self-monitoring logs, the therapist and patient collaboratively identified several barriers hindering progress. These barriers included the patient’s drive to “do more,” preoccupation with feelings of inadequacy regarding the role she could play in the climate crisis, uncertainty about the effectiveness of her actions in mitigating climate change, and distress about the uncontrollability of others’ behaviors contributing to ecological degradation. Primary resulting emotional states included guilt, anxiety, and panic. Concurrently, she experienced seasonal depression, coupled with feelings of hopelessness and despair fueled by recurrent thoughts of a dystopian future resulting from climate change.

Her low mood, heightened anxiety, and fluctuating self-worth contributed to urges to engage in restrictive and binge eating behaviors. Concerns about food waste and its environmental impact led to limited grocery purchases, preparing small meals to minimize food waste, and avoiding eating out to avoid non-recyclable packaging. Undernutrition from these behaviors had a notable effect on her energy and urges to binge eat. Further, the compulsion to prevent food waste by eating everything on her plate often resulted in eating beyond physical fullness, further exacerbating distress and increasing risk of binge eating and purging episodes.

Self-report measures were administered to obtain additional objective data on patient’s symptoms. The Patient Health Questionnaire-9 (PHQ-9; range 0–27) [21] and General Anxiety Disorder-7 (GAD-7, range 0–21) [22] assess depression and anxiety symptoms, respectively, and the EDE-Q [15] measures the frequency and severity of eating disorder symptoms; higher scores indicate greater severity. At the third month of treatment when eco-concerns worsened, the patient’s PHQ-9 score of 10 revealed moderate depression, a sharp increase from a score of 2 after the first month of treatment. In addition to self-reports of eco-anxiety, her GAD-7 score of 5 was in the mildly anxious range. According to these surveys, her problems with mood and anxiety made it “somewhat difficult” for her to do her schoolwork, take care of things at home, or get along with other people.

**Fig. 1:**
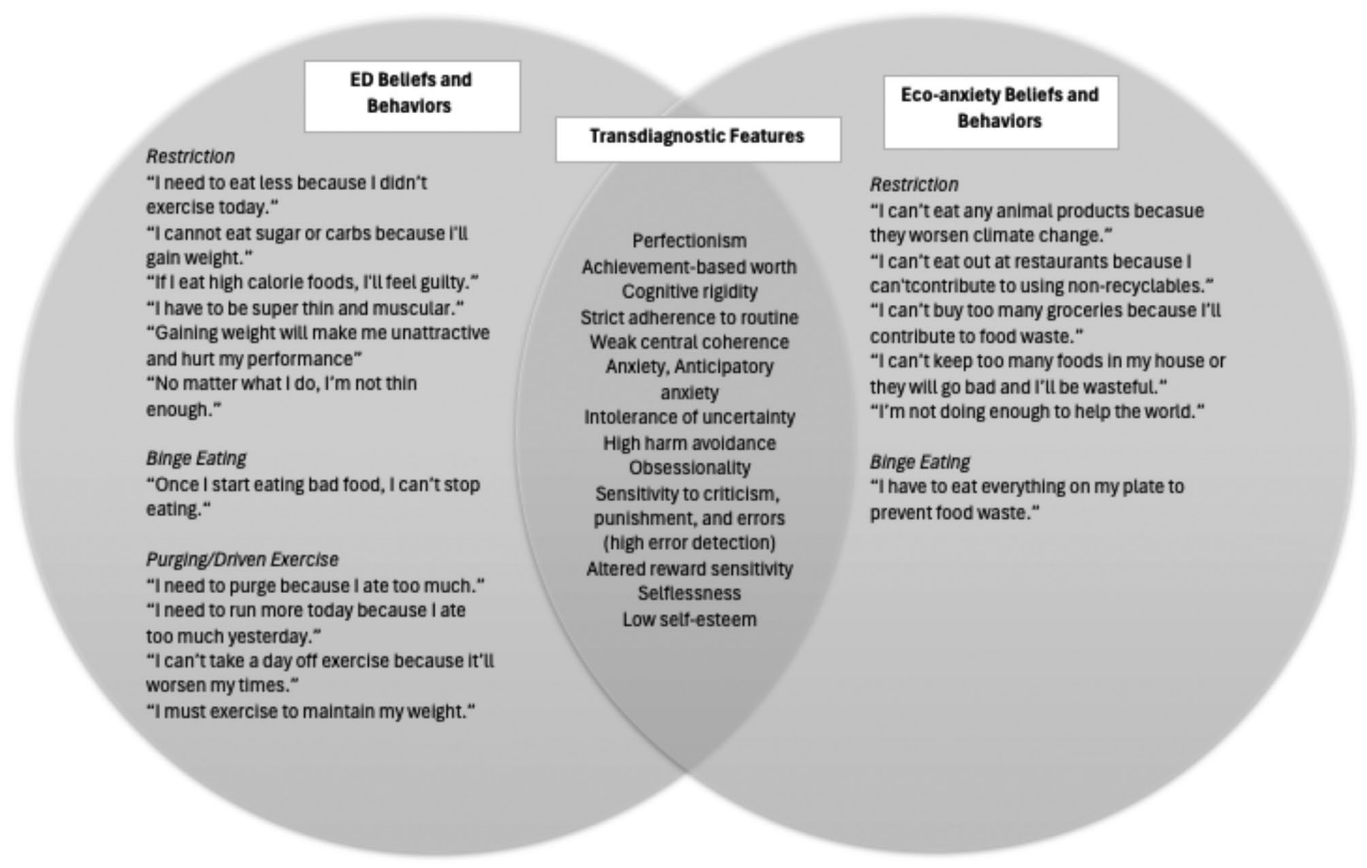
Transdiagnostic features of eating disorders and eco-anxiety. This Venn diagram provides case specific examples of *cognitions* leading to restriction, binge eating, and purging *behaviors*, stemming from either the eating disorder (left circle) or eco-anxiety (right circle). The intersection highlights key transdiagnostic features observed in the discussed case. For research supporting temperament traits in the etiology of eating disorders, refer to Hill, Knatz Peck, & Wierenga [23]

Due to the more prominent role of the patient’s eco-concerns in her unwillingness to engage in CBT-E challenges (e.g., ensuring adequate food supplies in her home), the primary therapist (CDR) conducted a literature review on eco-anxiety and consulted with a psychiatrist (DLS) specialized in climate change and mental health as well as eating disorders. In response to the patient’s interest in research, an article on eco-anxiety and eating disorders [11] was shared. This adaptation to the standard CBT-E psychoeducation impacted the patient and the subsequent course of treatment. The patient had not heard of eco-anxiety as a construct and felt validated that her symptoms had a name. She subsequently sought out additional information on the topic through podcasts. The therapist and patient used this newfound understanding of eco-anxiety to distinguish between dietary restriction driven by eco-concerns and that driven by the eating disorder itself. This distinction, coupled with the opportunity for the patient to express her emotions about climate change, proved critical in strengthening the therapeutic rapport, as the patient felt understood and respected. She trusted that the provider would not rigidly prescribe changes to eating that were not values-aligned. Importantly, this educational intervention increased the patient’s insight into the multifaceted nature of her eating disorder and its interaction with her eco-anxiety in exacerbating her symptoms. Shared mechanisms believed to underlie the two are outlined in Fig. 1.

In addition to the standard CBT-E food log, the therapist introduced thought monitoring on climate change concerns. This monitoring facilitated the identification of recurring cognitions contributing to distressing emotions, physical sensations, and behaviors. Together, the therapist and client separated distorted, unhelpful thoughts from realistic thoughts about ecological degradation. By exploring and restructuring certain automatic thoughts, such as “It’s not okay to feel happy because of everything going on in the world,” the patient challenged unhelpful cognitive biases and underlying assumptions. Collaboratively, the therapist and patient set goals to survey trusted climate change activists about their own thoughts and behaviors related to climate change, enabling the patient to recognize and counteract perfectionism as well as overly catastrophic and pessimistic thinking patterns. Encouraging the patient to spend more time with individuals concerned about climate change proved instrumental in broadening her perspective and fostering hope. Additionally, the effectiveness of regularly worrying about climate change efforts was explored. The patient grew increasingly motivated and willing to engage in more mindfulness practices to accept uncertainty about the future. This increased awareness of the dialectics in eco-anxiety (e.g., certainty vs. uncertainty, humanity’s strengths vs. weaknesses), helping her better contain difficult emotions and grief reactions related to eco-concerns.

Standard CBT-E techniques were also adapted to facilitate exploration of the short-term and long-term consequences of limiting her food purchasing and portion sizes. She gained insight into the adverse effects of food restriction on both personal health and her capacity for long-term engagement in activism efforts. Subsequently, she willingly engaged in a variety of in and out of session food-related exposures, including (1) increasing food accessibility in the home, (2) expanding portion sizes, (3) eating out more frequently, (4) packaging leftovers with reusable containers, and (5) discarding spoiled food without succumbing to the urge to overeat for fear of food waste.

Further, the patient and therapist examined the environmental impact of individual behavior change efforts in comparison with broader community and legislative efforts. This led her to recognize an unreasonable sense of personal responsibility and a better focus for her efforts on encouraging policy change. Like with achieving more balance with her marathon training (e.g., not overtraining, managing her energy and effort, resting before events), the patient was also able to see the importance of “pacing” herself in her individual efforts to reduce her carbon footprint. By aligning her individual actions with her values and receiving reinforcement from her climate change support network, she successfully integrated sustainable behavior changes into her lifestyle.

In parallel, general emotion management skills were emphasized, including pleasurable event scheduling (e.g., mindful walking in nature) and self-soothing techniques. Although not explicitly recommended in therapy, the patient began reducing exposure to media coverage of climate change (given these messages were overwhelmingly negative, both in terms of content and visually). She was reinforced for her use of previously-learned stimulus control strategies to reduce exposure to activating events in her environment.

Three months after identifying and discussing eco-anxiety and six months into CBT-E treatment, the patient was referred for a consultation to consider the addition of psychotropic medications given some lingering mood symptoms. She had never tried psychotropic medications and opted to defer a medication trial because she felt she was making progress in therapy to better make sense of her worsened mood, resulting in greater hope for improvement.

Following relapse prevention work, the patient and therapist collaboratively decided to terminate individual therapy.

By the conclusion of treatment, the patient achieved nutritional adequacy, ceased most maladaptive exercise, and no longer experienced regular urges to binge eat or purge. She remained vegan. EDE-Q^1^ scores were within the range of community norms for women aged 23–27 years [24], reflecting mild concerns regarding restraint, eating, shape, and weight. Although the EREC [14] was not completed during treatment, given the potential value of this measure, retrospective responses to the EREC for mid-treatment and post-treatment were gathered from the patient. The mid-treatment total score of 39 decreased to 32 by post-treatment. For comparison, the scores range from 10 to 46.

### Patient perspective

One year after treatment, the patient reported maintaining treatment gains and reflected on changes to her scores, stating:

I think the biggest takeaway from therapy / shift in climate-related anxiety about food was that I used to think a lot more about the amount of food I was consuming, along the lines of consuming less = lower environmental footprint. This led to restricting in the past. Additionally, with food waste, I previously would not waste food because more food waste = more emissions. This had led to binges when I wasn’t actually hungry. So kind of these two ends of the spectrum. Now, I think I am able to take a more holistic approach and fuel my body with what it needs and trying my best not to waste food but also not overeating in order to not throw away food.

## Discussion and conclusions

While research attention towards climate change’s impact on mental health is growing, there remains a notable dearth of knowledge concerning its specific influence on eating disorders. Rodgers and colleagues [11] proposed 4 primary pathways by which climate change may affect eating disorders: (1) decreased food access and security; (2) changes in mean temperature; (3) concerns related to food safety and eco-anxiety; and (4) indirect pathways through trauma, adversity, and increased mental health concerns. As Rogers and colleagues aptly articulate: “Given the climate benefits of environmentally sustainable diets, clarifying specific mechanisms and moderators of relationships between plant-based diets and eating disorder risk is critical, and longitudinal work is needed.”

This case report contributes empirical evidence supporting the third and fourth pathways connecting eco-anxiety to eating disorders, offering valuable insights for clinicians engaged in the evaluation and treatment of eating disorder patients. Specifically, this case underscores the importance of screening for concurrent eco-anxiety, suggests ways in which eating disorders and eco-anxiety can influence one another longitudinally, describes how coexisting eco-anxiety can limit the effectiveness of standard CBT-E, and provides examples of successful treatment adaptations tailored to address eco-anxiety-related concerns.

As noted, a key take-away from this case report includes the importance of assessing for eating-related eco-concerns and eco-anxiety in patients with disordered eating. Such assessment provides needed data for both the therapist and patient regarding if, or to what extent, eco-anxiety may be affecting the expression of the eating disorder. The present case report illustrates how therapists can seamlessly integrate screening questions into the initial inquiry of the patient’s motivation to avoid specific foods and adhere to a vegan diet. These queries unveiled the patient’s ethical apprehensions about consuming animal-based products (e.g., the environmental ramifications). Furthermore, an exploration of the patient’s educational background (e.g., her prior major in environmental science and related graduate work) and extracurricular activities revealed other non-food related proactive measures she took to protect the environment and slow climate change. Through self-monitoring records documenting triggers for depression and anxiety symptoms, a bidirectional relation between her mood symptoms and climate change concerns was identified within the first three months of treatment. Collectively, these data revealed the presence of eco-concerns as a driving force behind the patient’s dietary choices, distinct from being solely motivated by eating disorder cognitions. This case report highlights the importance of comprehensively assessing a patient’s underlying motivations for adopting vegetarianism or veganism and re-visiting this as needed.

Clinicians can reference Fuller and colleagues’ [25] suggested questions for help discerning whether a patient’s vegan diet primarily stems from ethical convictions or eating disorder-related motivations. These questions encompass observations of ethical practices beyond dietary choices, as well as the chronological sequence of dietary modifications. The specific questions are as follows: (1) Are ethical choices observable in non-food aspects of life, such as clothes, toiletries and use of free time? (2) Has there been a pattern of increasing dietary restriction, such as starting off with “healthy eating”, then vegetarianism and finally veganism, or were ethical concerns present before the dietary restriction began? In addition to using the EREC [14] to specifically evaluate eating-related eco-concerns, clinicians can employ other objective assessments to evaluate eco-anxiety more generally, such as the Hogg Eco-Anxiety Scale [26], the Climate Crisis Anxiety Scale [6], or the Climate Change Worry Scale [27].

This case also illustrated the importance of discussing the conceptualization of concurrent eating disorders and eco-anxiety with the patient. Doing so facilitated the patient’s understanding of the interconnections between her susceptibility to both an eating disorder and eco-anxiety, elucidating the underlying mechanisms common to both. For example, she was able to connect her anxious temperament, obsessionality, overvaluation of achievement, and cognitive rigidity in the development and perpetuation of her eating disorder. She also saw that these same pre-existing temperament traits, among others (see Fig. 1), heightened her vulnerability to experiencing eco-anxiety when studying the science of climate change. Relatedly, we conceptualized the eco-anxiety as primarily anxiety based and therefore more closely aligned with her generalized anxiety disorder than her depression. In addition to guiding the patient in drawing these parallels, the therapist validated the patient’s eco-anxiety, an important means of strengthening the therapeutic alliance. This process enabled the patient to experience sufficient distance to examine in what ways her behaviors could be best explained by her concerns for the environment and in what ways her behaviors were heavily influenced by her eating disorder and anxiety. Such distinctions were like those the patient had been able to make earlier in her life when she differentiated between athletic behaviors driven by aspirations for health and fitness, as opposed to those motivated by weight/shape concerns or anxiety and not helpful for her long-term well-being.

In the absence of data from longitudinal research on the development of eating disorders and eco-anxiety, details from this case report offer an example of the complex ways each can influence the development of the other. Examined longitudinally, the patient’s eating disorder was the first to develop and preceded the adoption of a vegan lifestyle. Veganism was primarily motivated by eco-concerns, although the extent of the eating disorder’s influence remained uncertain to the patient. Her eating disorder symptoms, as is often the case, recurred during a period of transition–in this instance her relocation for graduate school. Initially quickly responsive to CBT-E, her progress was impeded by worsening eco-anxiety (Roger’s third proposed pathway), which exacerbated eating disorder symptoms and hindered treatment progress. Integrating eco-anxiety as an additional treatment target likely impacted the duration of treatment to extend beyond the 20 sessions typically recommended in CBT-E for patients who are not significantly underweight [20]. The longitudinal trajectory of the patient’s symptoms was further complicated by the patient’s mood, anxiety disorders, and personality traits (Roger’s fourth proposed pathway). As such, as noted by Rodgers et al. [11], there are important mechanisms underlying these constructs that need to be considered in treatment and research.

Until her eco-anxiety was fully recognized and addressed, these concerns posed an obstacle to sustained progress in her CBT-E treatment. In summary, successful adaptations to CBT-E interventions targeting eco-anxiety used with the discussed case included: education about climate change and mental health; expanding the conceptualization of eating disorder’s influence on restrictive behaviors to include eco-anxiety; providing a safe container for emotions related to climate change, including validation for these emotional states; use of thought monitoring and Socratic questioning as well as food-related exposures (e.g., increasing food accessibility in the home) to identify and intervene on unhelpful cognitions and behaviors related to climate change; emotion regulation skills (e.g., mindful walking in nature); stimulus control to reduce exposure to overly negative messages and vivid imagery regarding climate change; encouragement to receive emotional support from other climate change activists; identification of the pros and cons of solely focusing on individual response efforts as opposed to attention to the power of community-level actions and legislative efforts; and creating sustainable changes to her lifestyle.

These interventions are consistent with those identified by systematic and scoping reviews of interventions targeting eco-anxiety [28, 29]. Although only two interventions for eco-anxiety have undergone preliminary testing, qualitative studies outlining provider perspectives on eco-anxiety treatment consistently highlight key themes–such as emotion regulation, problem-focused coping, social support, connection with nature, and resiliency–that are deemed vital for effectively addressing eco-anxiety broadly. Of note, therapists are cautioned against rushing patients too quickly into action without first addressing the understandable emotional hardships faced from climate change [30, 31]. Lessons learned from this case report and recommendations to treating providers of patients with eating disorders are listed in Table 1.

**Table 1:** Lessons Learned/Recommendations*

● Be an eco-aware provider, able to demonstrate knowledge and understanding of climate change’s impacts on the environment and individual emotional well-being. This encompasses the capacity to provide psychoeducation on eco-anxiety and its effects on mental health when needed. ● Recognize the particular importance of assessing for eco-anxiety in patients with eating disorders who adhere to a vegan or vegetarian diet. ● Create space for patients to share their eco-concerns openly and to validate these concerns. This space may improve the alliance, building trust as well as aid in emotion regulation. ● When possible, use the EREC to identify restricted eating patterns motivated by eco-related concerns. Recognizing that restriction can be multi-determined may loosen the patient’s insistence on denying any role of eating-disorder-related motivations. ● Relatedly, while it is important to recognize that weight and shape concerns may not be the sole drivers of restrictive eating patterns, be aware that eco-related eating concerns may mask co-existing or primary eating disorder motivations. ● When eco-anxiety is prominent, CBT may be used to address impairing eco-anxiety symptoms. ● In addition, eco-anxiety can be managed by building media literacy. This may include assisting patients in adjusting their media consumption of climate change material, particularly by reducing exposure to overly negative or overwhelming messaging. ● Strongly encourage expanding the patient’s social support, such as through established groups and organizations (e.g., climate cafes https://www.climate.cafe/ ). ● Be aware of the variables that can increase the risk of experiencing eco-anxiety ○ Age: Adolescents and young adults ○ Focus of study or work: Majoring in environmental studies, working or volunteering as a climate activist ○ Co-morbid disorders: e.g., depression, anxiety ○ Pre-existing temperament traits (see Fig. 1).	

*Given the nature of this case report, this list of options for supporting patients dealing with impairing eco-anxiety is not exhaustive. Readers are referred to Baudon & Jachens [27], Xue et al. [32], and Wilkinson & Wray [33] for additional recommendations stemming from the qualitative and preliminary work on general interventions for eco-anxiety

Although this case report offers insights into how eco-anxiety can present and be treated in a patient with a pre-existing eating disorder, it, like all case reports, is subject to several limitations. For example, findings may not generalize to other patients and are vulnerable to reporting bias. To mitigate bias, we extracted data from the patient’s medical chart, including two psychiatric evaluations by CDR and DLS as well as clinical notes, and reported on validated objective measurement results. However, we lacked objective assessment data on climate change concerns pre- and post-treatment as well as at follow-up. While the patient retrospectively responded to a validated measure on eating-related eco-concerns (i.e., the EREC), this approach is susceptible to recall bias. Additionally, the absence of a control group prevents us from determining whether observed outcomes are attributable to the treatment described or influenced by other factors.

Longitudinal studies are needed to delineate the complex interplay between eating disorders and eco-anxiety. Additionally, larger scale intervention studies with diverse samples are needed to determine effective interventions tailored to address eco-anxiety in the context of eating disorders. For example, future research may compare adapted versions of current evidence-based treatments with traditional versions. In their urgent call for research on climate change and eating disorders, Rodgers et al. [11] propose multiple other important avenues for research.

To our awareness, this is the first case report demonstrating the way eco-anxiety can intersect with eating disorders to affect treatment effectiveness. Eco-anxiety, a relatively newly-described syndrome, involves the fear of environmental doom [5]. Symptoms of eco-anxiety span several domains such as obsessive rumination about the future, distressing emotions (e.g., fear, dread, anger, grief, guilt), and difficulty concentrating [6]. Despite the potential clinical concerns associated with concurrent eco-anxiety and eating disorders, the relation between the two constructs has received very little attention. Rodgers and colleagues [11] have highlighted this research gap, emphasizing the need for investigations on how climate change may influence the onset, maintenance, or treatment outcomes of eating disorders. This case report responds to their call, providing an initial step toward understanding this relationship. In particular, this case report contributes empirical evidence supporting two of Rodgers et al.’s four proposed pathways connecting climate change with eating disorders, i.e., through (1) eco-anxiety and (2) increased mental health concerns. As such, this report underscores the importance of more rigorous research explicating the association between eating disorders and climate change. Additional research is needed to test clinical interventions designed to treat concurrent eating disorders and eco-anxiety.

## Data Availability

No datasets were generated or analysed during the current study.

## Footnotes

1 EDE-Q scores [in comparison with community norms (M, SD) for women aged 23–27 years] [24]: 1.2 (M 1.34, SD 1.39) restraint, 0.8 (M 0.81, SD 1.10) eating concern, 1 (M 2.24, SD 1.61) shape concern, 0.6 (M 1.84, SD 1.50) weight concern, 0.9 (M 1.56, SD 1.26) global score.

## Abbreviations

BMI: Body mass index
CBT-E: Cognitive Behavior Therapy-Enhanced
DSM-5TR: Diagnostic and Statistical Manual of Mental Disorders, Fifth Edition, Text Revision DSM-5-TR^®^
EDE-Q: Eating Disorder Examination-Questionnaire
EREC: Eating-Related Eco-Concern Questionnaire
GAD-7: General Anxiety Disorder-7
PHQ-9: Patient Health Questionnaire-9
